# Parliament and the Profession

**Published:** 1918-03-30

**Authors:** 


### PARLIAMENT AND THE PROFESSION.

### Officers' Artificial Limbs.

The Minister of Pensions informed Colonel Ashley that an officer of the Army who loses a leg is fitted with an artificial one free of cost, and is given a wound pension of £100 a year, out of which he is expected to defray the expense of repair and renewal. Mr. Hodge added that, in view of misleading statements which have appeared in the press, it should be added that a wound pension is in addition to retired pay, and that no officer who loses a leg in action gets less, in the aggregate, than £150 a year. The question whether it is possible to give military officers further assistance in the matter of artificial limbs is being considered.

### Canadian Red Cross.

Sir Alfred Mond informed Lord Henry Cavendish-Bentinck, who asked a question as to the taking over of the Canadian Red Cross quarters in Cockspur Street for the purposes of the Ministry of Munitions, that it was only after the most careful consideration by the War Cabinet Committee on Accommodation that the step was taken. The Canadian Red Cross have been consulted throughout, and new and commodious premises are being acquired for them.

